# Physicochemical Characterization of Anionic Lipid Membranes under Low and Physiological Ionic Strength: Effects of Moxifloxacin Assessed by Calorimetry, Spin-Label, Steady-State, and Time-Resolved Fluorescence

**DOI:** 10.1021/acsomega.5c11663

**Published:** 2026-06-10

**Authors:** Arthur Henrique Barrios Solano, Mariana C. Souza, Carla C. V. Medeiros, Arthur S. Borges, Bruno Sugamosto, Evandro L. Duarte, M. Teresa Lamy, Gabriel S. Vignoli Muniz

**Affiliations:** † Instituto de Química, 28127 Universidade de Brasília, Campus Universitário Darcy Ribeiro, 70910-900 Brasília, Brasil; ‡ Instituto de Física, Universidade de São Paulo, Cidade Universitária, 05508-090 São Paulo, Brasil

## Abstract

In the present study, we comparatively investigate the interaction between the antibiotic moxifloxacin (MFX) and extruded (100 nm) lipid vesicles composed of the anionic lipid 1,2-dipalmitoyl-*sn*-glycero-3-phospho-(1′-rac-glycerol) (DPPG), under both low ([NaCl] = 3 mmol L^–1^) and physiological ionic strength conditions ([NaCl] = 150 mmol L^–1^). By employing differential scanning calorimetry (DSC) and electron spin resonance (ESR) spectroscopy with spin-labeled lipids positioned at two distinct depths within the bilayer, we evaluated structural alterations induced by increasing concentrations of MFX. Additionally, taking advantage of the intrinsic fluorescence of the antibiotic, we monitored structural changes and determined its affinity for the anionic bilayers upon exposure to rising concentrations of liposomes under different configurations: gel and fluid phases. MFX was found to induce a narrowing of the main gel-to-fluid phase transition peak of DPPG under both ionic strengths, indicating enhanced cooperativity of the thermal lipid transition. ESR spectra of spin labels inserted at the fifth carbon position, near the interfacial region between the polar headgroups and the hydrophobic core, and deeper within the bilayer revealed that MFX promotes tighter lipid packing and decreases the local polarity of the membrane interior, independently of salt concentration. Upon increasing the concentration of anionic liposomes, the MFX emission displays a relative redshift. The data suggest that, upon interaction with the anionic bilayers, a fraction of the zwitterionic MFX molecules undergoes protonation, presumably driven by a locally low pH microenvironment at the vesicle interface. MFX fluorescence lifetimes are enhanced by its association with gel and fluid liposomes, enabling the determination of membrane-water partition coefficients, which were found to be *K*_p_ = (2.5 ± 0.2) × 10^4^ and (1.4 ± 0.1) × 10^4^ for the gel and fluid phases, respectively, under low ionic strength conditions. Furthermore, given the relevance of antibiotic–membrane interactions to antimicrobial efficacy, toxicity, and drug delivery strategies, the findings reported herein advance the understanding of the physicochemical behavior of MFX in lipid environments and may guide the rational development of membrane-targeting antibiotics and lipid-based delivery systems.

## Graphical abstract

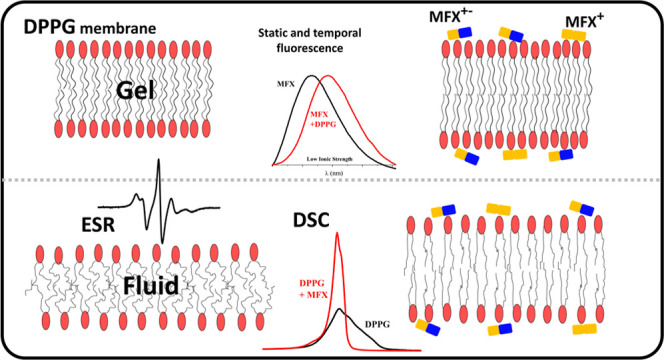

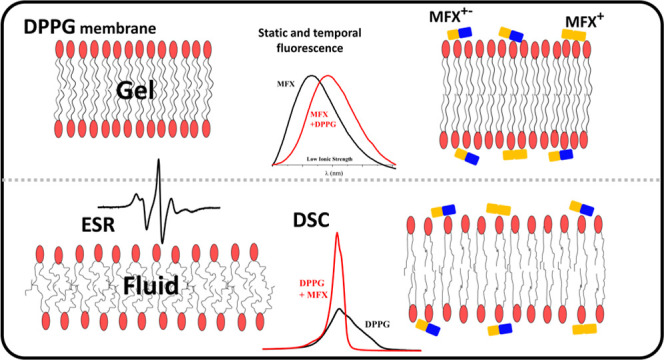

## 1 Introduction

Fluoroquinolones (FQs) are a widely used class of antibiotics in both human and veterinary medicine. Their well-established mechanism of action involves the inhibition of the bacterial enzymes topoisomerase II and IV, through the formation of stable drug–enzyme–DNA complexes.^1^ Consequently, FQs must reach the bacterial cytosol to exert their antimicrobial effects. To penetrate the cytosol of Gram-negative bacteria, many fluoroquinolones rely on porin channels to cross the outer membrane.^1,2^ Particularly, 1-cyclopropyl-7-(2,8-diazabicyclo[4.3.0]­non-8-yl)-6-fluoro-8-methoxy-4-oxoquinoline-3-carboxylic acid, better known as moxifloxacin (MFX; Figure 1), is a potent FQ with activity against both Gram-positive and Gram-negative bacteria. Regarding its uptake in Gram-negative bacteria, MFX appears to have little dependence on porin channels, suggesting that interactions between this antibiotic and membrane lipids may play an important role in facilitating its access to the cytosol.^3,4^

**Figure 1:**
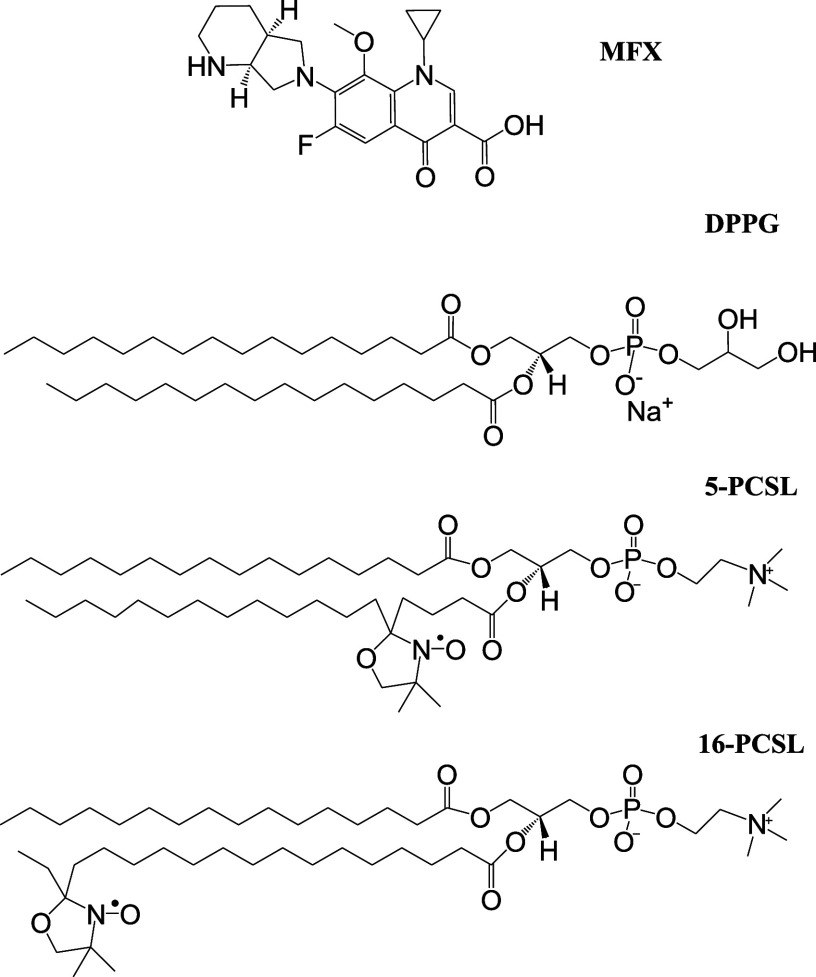
At the top, schematic chemical structure of the antibiotic moxifloxacin. At the bottom, the chemical structures of the anionic lipid DPPG, and the paramagnetic probes 5-PCSL and 16-PCSL.

Studies with MFX and model membranes have shown that the antibiotic interacts with lipids. High concentrations of MFX induce dramatic structural alterations in extruded lipid dispersions composed of pure 1,2-dipalmitoyl-*sn*-glycero-3-phosphocholine (DPPC) and DPPC/cardiolipin (CL) mixtures (8:2 molar ratio), using a lipid-to-antibiotic molar ratio of -approximately 1:1.^5^ Also, the interaction between MFX and zwitterionic membranes composed of 1,2-dimyristoyl-*sn*-glycero-3-phosphocholine (DMPC) or 1,2-dimyristoyl-*sn*-glycero-3-phosphorylglycerol (DMPG), was investigated showing that MFX has much higher affinity for anionic liposomes, fluorescence probes and spin labels embedded into lipid bilayers strongly indicates that MFX acts in the surface of anionic DMPG vesicles.^6^

Phosphatidylglycerol (PG) is the most abundant anionic lipid in bacterial membranes.^7^ As such, it has become a frequent target in the development of antimicrobial drugs.^7,8^ Moreover, the interaction of drugs with anionic lipids may plays a key role in their ability to penetrate bacterial membranes and reach the cytosol, as observed in the case of moxifloxacin (MFX).^4^ PG lipids have also been explored in the formulation of drug delivery systems due to their biocompatibility and functional properties.^9^

To better understand the interaction between MFX and anionic lipids, and to provide insights that may aid in the design of more effective drug delivery systems, we investigated the interaction of MFX with 1,2-dipalmitoyl-*sn*-glycero-3-phospho-(1′-rac-glycerol) (DPPG) liposomes. We employed two distinct liposome configurations: one in the gel phase, representing more ordered and tightly packed membrane regions, and another in the fluid phase, mimicking more disordered and dynamic areas of bacterial membranes. As an initial step, we focused on liposomes composed of a single lipid component to reduce complexity and generate a solid basis for interpreting interactions in more complex lipid systems in future studies.

In this work, we monitored liposome structural properties using differential scanning calorimetry (DSC) and electron spin resonance (ESR) with spin probes inserted at different depths of the bilayers. The antibiotic’s behavior and environment were assessed via its intrinsic fluorescence, using both steady-state and time-resolved fluorescence spectroscopy. Considering that liposome structure and drug–lipid interactions are influenced by ionic strength,^10,11^ all experiments were performed under both low ionic strength ([NaCl] = 3 mmol L^–1^) and physiological ionic strength conditions ([NaCl] = 150 mmol L^–1^). Thus, this work contributes to a deeper understanding of how antibiotics relate to lipid assemblies, providing information that may support the development of more efficient and targeted drug-delivery systems, while also offering a clearer view of the mechanisms governing the action and cellular uptake of MFX across bacterial membranes.

## 2 Materials & Methods

### 2.1 Chemical and Reagents

Sodium salt of 1,2-dipalmitoyl-*sn*-glycero-3-phospho-(1′-rac-glycerol) (DPPG), spin labels 1-palmitoyl-2-(*n*-doxylstearoyl)-*sn*-glycero-3-phosphocholine (*n*-PCSL, *n* = 5 or 16) were acquired from Avanti Polar Lipids. Moxifloxacin (MFX), 4-(2-hydroxyethyl)-1-piperazineethanesulfonic acid (HEPES), ethylenediaminetetraacetic acid (EDTA), boric acid, phosphoric acid 85%, citric acid, sodium hydroxide (NaOH), hydrochloric acid (HCl), chloroform, methanol, and sodium chloride (NaCl) were purchased from Merck (St Louis, MO). All solutions or dispersions were prepared using Milli-Q water or a mixture of chloroform and methanol.

### 2.2 Sample Preparations

Lipids were initially solubilized in a chloroform/methanol mixture (6:1, v/v). For ESR measurements, the spin probes 5-PCSL (0.8 mol %) or 16-PCSL (0.3 mol %) were incorporated into the lipid solution, with molar fractions defined relative to the total lipid content. The solvent was subsequently removed under a gentle stream of ultrapure nitrogen, leading to the formation of a thin lipid film on the walls of a glass tube. This film was further subjected to vacuum for at least 3 h to ensure complete removal of residual organic solvents. The buffers used in this study consisted of 10 mmol L^–1^ HEPES, 1 mmol L^–1^ EDTA, pH 7.4, with either 3 mmol L^–1^ NaCl (low ionic strength) or 150 mmol L^–1^ NaCl (physiological ionic strength). For the determination of MFX p*K*_a_ values, a universal buffer was used, composed of a mixture of 33 mmol L^–1^ citric acid, 50 mmol L^–1^ phosphoric acid, 50 mmol L^–1^ boric acid, and 330 mmol L^–1^ NaOH in its stock solution. The universal buffer was prepared by diluting the stock buffer at a ratio of 1:15 in water and adjusting the pH using a 1 mol L^–1^ HCl solution.^12^

Lipid dispersions were obtained by hydrating the dry lipid film with the appropriate buffer, followed by vigorous vortexing (2 min at 60 °C), repeated four times. The resulting suspensions were extruded through polycarbonate membranes (100 nm pore size, 31 passes) using a mini-extruder (Avanti Polar Lipids), at temperatures above the gel–fluid transition (≥60 °C), yielding large unilamellar vesicles (LUVs). All dispersions were freshly prepared on the day of use.

MFX stock solutions (6 mmol L^–1^ in methanol) were prepared, and aliquots were dried under nitrogen to form a homogeneous antibiotic film. Residual solvent was removed under vacuum (≥3 h). Hydration was then performed using buffer or preformed LUV dispersions, followed by vortexing (2 min at 60 °C, four cycles). Samples were equilibrated for 30 min at room temperature prior to analysis. The hydrodynamic diameter (*D*_z_) of vesicles, in the absence and presence of MFX, was determined by DLS. No significant variation in vesicle size was observed, with *D*_z_ values around 100 nm and low polydispersity under both ionic strength conditions, as shown in Table S1. Under both low and physiological ionic strength conditions, vesicles in either the gel or fluid phase exhibited *D*_z_ values of approximately 100 nm, with a low polydispersity index (PDI) (Table S1).

### 2.3 Differential Scanning Calorimetry (DSC)

DSC experiments were carried out using a MicroCal VP-DSC microcalorimeter. Each sample underwent five consecutive scans; the first (90 °C h^–1^) was used to erase thermal history and excluded from analysis. Subsequent scans (two heating and two cooling) were performed at 20 °C h^–1^ over the range 10–60 °C and exhibited good reproducibility. The sample cell (500 μL) contained lipid dispersions (3 mmol L^–1^) with or without MFX. Antibiotic concentration was expressed as molar percentage relative to lipid content (mol % = 100­[MFX]/[L]). Thermodynamic parameters such as transition temperature (*T*_m_), enthalpy change (Δ*H*), and transition width (Δ*T*_1/2_) were obtained using MicroCal Origin software.

### 2.4 Electron Spin Resonance (ESR) Spectroscopy

ESR spectra were recorded on a Bruker EMX spectrometer (X-band), using modulation amplitudes of 1–2 G and microwave power of 13 mW. Temperature control (10–60 °C) was achieved with a Bruker BVY-2000 unit. ESR Spectra correspond to the first derivative of microwave absorption with respect to magnetic field and were normalized using the central peak. For 5-PCSL, outer (2 *A*_max_) and inner (2 *A*_min_) splittings were directly measured, and, in fluid phases, *S*_eff_ and *a*_0_ were calculated according to eq 1

1 $$S_{eff}=\frac{A_{∥}-A_{⊥}}{A_{zz}-\frac{1}{2}\left(A_{xx}+A_{yy}\right)}\frac{a_{o}^{\prime }}{a_{o}}$$

where

$$A_{∥}\approx A_{\max}$$

$$A_{⊥}=A_{\min}+1.4[1-\frac{A_{∥}-A_{\min}}{A_{zz}-\frac{1}{2}\left(A_{xx}+A_{yy}\right)}]$$

$$a_{0}^{\prime }=\frac{1}{3}\left(A_{zz}+A_{yy}+A_{xx}\right)$$

$$a_{0}=\frac{1}{3}\left(A_{∥}+{2A}_{⊥}\right)$$

in which *A*_*zz*,_ *A*_*xx*_, and *A*_*yy*_ are the principal values of the hyperfine tensor for 2-doxyl propane, 32.9, 5.9, and 5.4 G, respectively.^13,14^ For the 16-PCSL probe, the central line width (Δ*H*_0_) was evaluated under gel-phase conditions. In the fluid phase, the analysis was based on the relative amplitudes of the hyperfine components (*h*_+1_, *h*_0_, and *h*_–1_), together with the isotropic hyperfine splitting (*a*_0_), all extracted directly from the ESR spectra. Further methodological details are provided in ref 15. The lipid concentration used was 3 mmol L^–1^, with and without the desired MFX concentrations.

### 2.5 Ultraviolet–Visible (UV–Vis) Absorption Spectroscopy

Optical absorption spectra were recorded using a UV–vis spectrophotometer (Varian Cary 50, Santa Clara, CA). In all optical experiments, briefly outlined here, the procedure followed the protocol previously established in our earlier studies (refs 16–18 ). Samples were placed in quartz cuvettes (0.2 × 1.0 cm, 400 μL), with an optical path length of 0.2 cm. Temperature control was achieved using a Cary Peltier thermostat.

Samples were prepared as follows. MFX films (see Section 2.2) were hydrated with buffer to obtain a stock solution at 0.5 mmol L^–1^. This stock was subsequently diluted using the same buffer and/or concentrated LUV dispersions (10 mmol L^–1^, see Section 2.2) to reach a final MFX concentration of 8 μmol L^–1^, in the absence or presence of the desired lipid concentration. The resulting samples were vortexed briefly and allowed to equilibrate at room temperature for 5 min.

Measurements were first carried out at 25 °C, corresponding to the gel phase of DPPG bilayers. After insertion into the spectrophotometer, samples were left for an additional 5 min to ensure thermal equilibration. Optical measurements were performed sequentially: absorption spectra were acquired first, followed by steady-state fluorescence and, subsequently, time-resolved fluorescence measurements.

Subsequently, the temperature was increased to 50 °C, corresponding to the fluid phase of dipalmitoyl bilayers, and the same experimental sequence was repeated under these conditions.

### 2.6 Stead-State Fluorescence Spectroscopy

Steady-state fluorescence experiments were conducted on a Varian Cary Eclipse spectrofluorimeter (Santa Clara, CA), with temperature maintained by a Peltier-controlled system. Measurements were performed using 400 μL samples containing MFX (8 μmol L^–1^) in buffer, either in the absence or in the presence of LUV dispersions, as previously described.

Excitation was performed at 345 nm using a reduced optical path length of 0.2 cm. The excitation and emission bandwidths were both set to 5 nm. All fluorescence emission spectra were corrected for inner filter effects^19^ according to eq 2

2 $$F_{corr}\left(\lambda \right)=F_{obs}\left(\lambda \right)10^{\left(A_{exc}l+A_{ems}\left(\lambda \right)l^{\prime }\right)}$$

where *F*_corr_(λ) and *F*_obs_(λ) are the corrected and observed fluorescence intensities, *A*_exc_ and *A*_ems_(λ) are the absorbances per unit of pathway at the excitation and emission wavelengths, respectively, and *l* and *l*′ are the optical pathways for excitation (0.1 cm) and emission (0.5 cm), respectively, considering the cuvette center.

### 2.7 Time Resolved Fluorescence Spectroscopy

Time-resolved (TR) fluorescence measurements were carried out using the time-correlated single photon counting (TCSPC) technique. The excitation source consisted of a titanium–sapphire laser (Tsunami 3950, Spectra Physics, Newport Corporation, Irvine, CA, USA), pumped by a Millenia Pro J80 solid-state laser. A pulse picker (Spectra Physics, model 3980-25) was operated at 8 MHz. The fundamental output of the Tsunami laser (852 nm) was converted to 284 nm through third-harmonic generation using a BBO crystal (GWN-23PL, Spectra Physics).

Although this excitation wavelength differs from that used in steady-state fluorescence experiments (345 nm), it lies within an absorption band of MFX. Importantly, the fluorescence relaxation dynamics of MFX were found to be independent of the excitation wavelength (Figure S1). The use of 284 nm significantly improved the signal-to-noise ratio in the time-resolved measurements compared to excitation at 345 nm. By using FAST software supplied by Edinburgh Photonics the data were fitted by applying the model of exponential decays^20^ using the following eq 3

3 $$F\left(\lambda ,t\right)=\sum_{i=1}^{N}\alpha_{i}e^{-t/\tau_{i}}$$

where *F*(λ,*t*) is the number of photons emitted at a given wavelength (λ) and time, *t* is the time after the excitatory light beam, α_*i*_ is the pre-exponential factor, τ_*i*_ is the lifetime of the *i*th component of the decay, and f_*i*_ is the fraction contribution of the lifetime τ_*i*_ to the intensity decay. All the fluorescence decay curves were well fitted by a biexponential model, as indicated by the reduced chi-square (χ^2^) statistical parameter, 0.80 ≤ χ^2^ ≤ 1.33, and resulting in two fluorescence lifetimes a short lifetime (τ_1_) and a longer lifetime (τ_2_).

The apparent partition constant (*K*_p_) was obtained through nonlinear fitting using a conventional isotherm,^21^ according to eq 4.

4 Kp≡nLVLnaqVaq

$$\tau_{2}=\frac{\tau_{2w}+K_{p}\gamma_{L}\left[L\right]\tau_{2L}}{1+K_{p}\gamma_{L}\left[L\right]}$$

where *n*_L_ and *n*_aq_ are the numbers of moles of MFX in the membrane and aqueous phases, respectively; *V*_aq_ is the volume of the aqueous phase, which is in a good approximation equal to the total volume (*V*_total_), taking into consideration both phases, aqueous and lipid; *V*_L_ is the volume of the lipid phase, *V*_L_ = γ_L_[L]*V*_total_; γ_L_ is the molar volume of DPPG lipids, for which values were taken from literature data, yielding 0.67 L mol^–1^ for gel-phase DPPG membranes and 0.71 L mol^–1^ for fluid-phase membranes;^22^ τ_2_, τ_2w_, and τ_2L_ correspond to the longer fluorescence lifetime of MFX at a given lipid concentration, in the absence of lipids, and under lipid-saturation conditions, respectively.^23^

### 2.8 Reproducibility and Sample Stability

Each experiment was performed in triplicate. The values display in this work consist of an average of the measurements and error values account for standard deviations and are presented as error bars when larger than the symbols. No vesicle precipitation was observed during the experiments. The samples were always visually checked before and after the measurements were taken. For optical absorption and fluorescence measurements, MFX spectra were quite reproducible after a minimum of 3 h in the cuvette.

## 3 Results and Discussion

### 3.1 Liposome Modifications Caused by Moxifloxacin

Liposomes, commonly composed of a single saturated lipid species, often exhibit two distinct thermal phases: a gel phase and a fluid phase. In both phases, the lipids are arranged within the two-dimensional plane of the bilayer. However, lipid mobility differs significantly between these phases. In the gel phase, lipids are organized in a manner that displays lateral and crystalline order.^24^ Conversely, in the fluid phase, lipids lack this order and exhibit greater movement along the axis of the paraffinic chains, displaying a more isotropic motion.^24^

Exogenous molecules can interact differently with gel-phase and fluid-phase liposomes. To investigate this, we examined the interactions between MFX and DPPG bilayers in their gel and fluid phases, mimicking lipid domains with tighter packing and greater fluidity, respectively, as observed in biological membranes. The structure and dynamics of acidic lipids, as well as their interactions with foreign molecules, are often influenced by ionic strength conditions.^25,26^ Thus, we analyzed the interaction between the antibiotic and DPPG dispersions under low ([NaCl] = 3 mmol L^–1^) and physiological ([NaCl] = 150 mmol L^–1^) ionic strength conditions.

#### 3.1.1 Differential Scanning Calorimetry (DSC)

The lipid gel–fluid phase transition is a phenomenon heavily reliant on interactions among lipid molecules and their surrounding environment. As a result, the phase transition can be influenced by factors such as pH, ionic strength, and the presence of foreign molecules, rendering it sensitive to external influences.^27^

Thermal analysis through DSC traces, spanning temperatures from 10 to 60 °C, provides valuable information into the thermal traits of DPPG membranes and the alterations induced by MFX in the DPPG structure. Figure 2 exhibits DSC traces of extruded (100 nm) DPPG dispersions under low (Figure 2 left column) and physiological ionic strength conditions (Figure 2 right column). The investigations include conditions in the absence of MFX (Figure 2a, b) and in the presence of 10 mol % MFX (Figure 2c, d) and 20 mol % MFX (Figure 2e, f).

**Figure 2:**
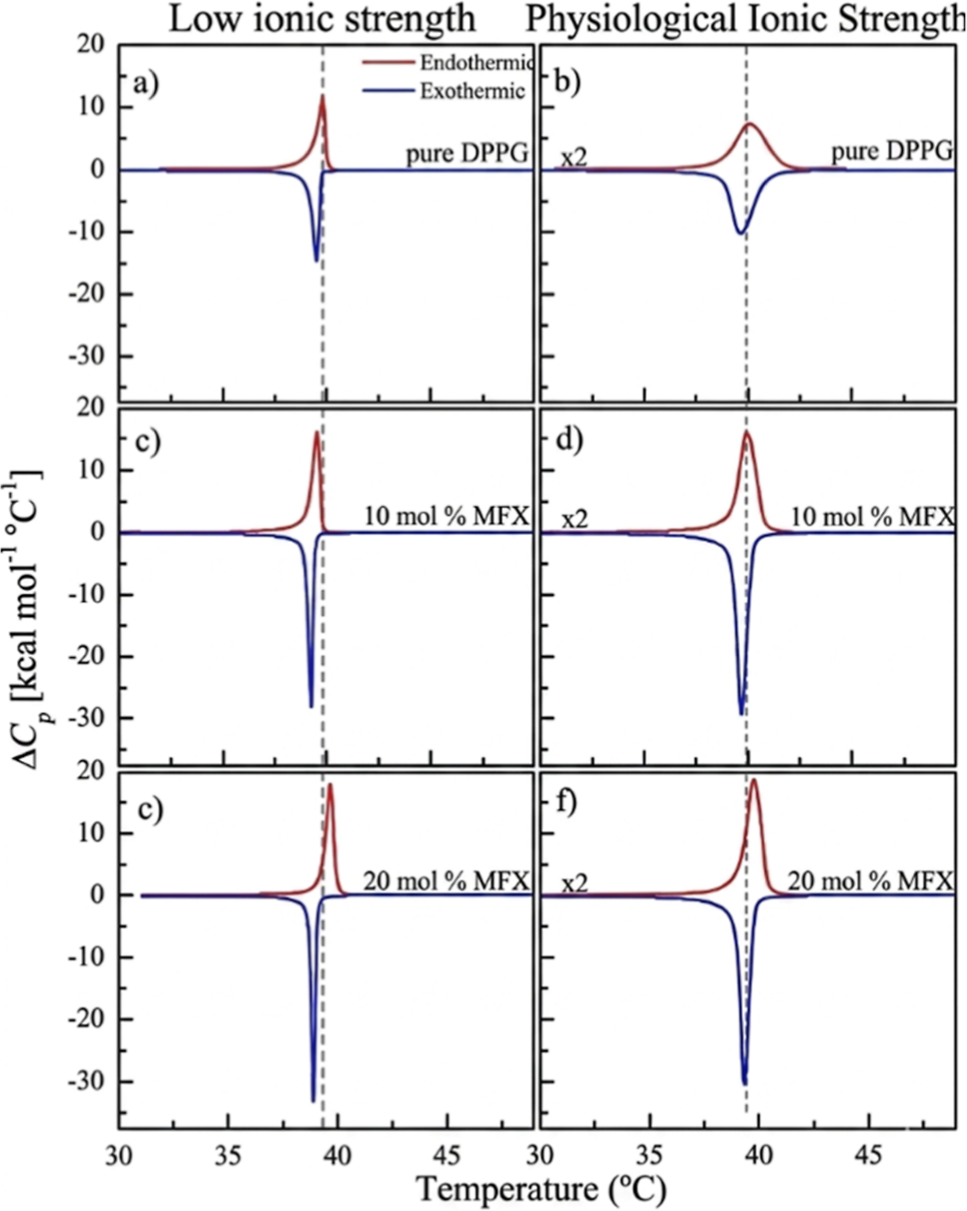
Typical DSC traces of 3 mmol L^–1^ DPPG dispersions in buffer at low ionic strength ([NaCl] = 3 mmol L^–1^, left column) and in at physiological ionic strength ([NaCl] = 150 mmol L^–1^, right column) in the absence (a,b) and in the presence of increasing amounts of MFX: 10 mol % (c,d), and 20 mol % (e,f) under endothermic (red lines) and exothermic (blue lines) processes. The dotted lines are only a guide for the eyes and indicate the positions of the maximum of the thermal gel–fluid transition peak.

In both dispersions, under both low (Figure 2a) and physiological ionic conditions (Figure 2b), the DSC traces consistently displayed thermal peaks centered at identical positions (*T*_m_), as indicated by overlapping standard deviation in Table 1. A similar behavior was reported by Riske et al.,^11^ who observed only a minor shift in the *T*_m_ (∼0.3 °C) of nonextruded DPPG dispersions when the NaCl concentration was varied from 2 mmol L^–1^ to 100 mmol L^–1^. Consistent with these observations, our results show that increasing the NaCl concentration from 3 mmol L^–1^ to 150 mmol L^–1^ does not produce a measurable change in *T*_m_ within experimental error (see Table 1). Although the absolute salt concentrations differ slightly between the two studies, the comparable ionic strength ranges support the conclusion that, at physiological pH, the main phase transition temperature of anionic lipid bilayers is largely insensitive to ionic strength within this range of NaCl concentrations. The enthalpy changes (Δ*H*) of MLV and LUV DPPG dispersions are very similar (≈8.6 kcal·mol^–1^),^11,28^ in good agreement with the values obtained in our study. Interestingly, the presence of MFX, under both low and physiological ionic strength conditions, slightly increases the Δ*H* values. This behavior may indicate that MFX promotes tighter lipid packing and/or partial dehydration of the DPPG vesicles, thereby strengthening tail–tail interactions. As a consequence, a higher amount of energy is required to drive the gel-to-fluid phase transition.

**Table 1:** Summarizes the Thermodynamic Parameters Determined through DSC Traces

		*T*_m_ (°C)		Δ*H* (kcal mol^–1^)		Δ*T*_1/2_ (°C)	
[NaCl] (mmol L^–1^)	mol % [MFX]	endothermic	exothermic	endothermic	exothermic	endothermic	exothermic
3	0	39.8 ± 0.2	39.5 ± 0.1	8.8 ± 0.3	–8.4 ± 0.3	0.57 ± 0.09	0.44 ± 0.06
150	0	39.8 ± 0.3	39.5 ± 0.1	9.8 ± 0.9	–9.5 ± 0.8	1.5 ± 0.5	1.3 ± 0.1
3	10	39.5 ± 0.4	38.8 ± 0.5	10.3 ± 0.1	–9.65 ± 0.05	0.42 ± 0.03	0.23 ± 0.01
150	10	39.8 ± 0.4	39.6 ± 0.1	10.0 ± 0.4	–9.2 ± 0.3	0.92 ± 0.04	0.50 ± 0.07
3	20	39.70 ± 0.06	38.88 ± 0.04	9.9 ± 0.2	–8.9 ± 0.2	0.33 ± 0.02	0.16 ± 0.01
150	20	39.8 ± 0.1	39.32 ± 0.04	10.9 ± 0.7	–9.3 ± 0.3	0.83 ± 0.07	0.435 ± 0.005

Moreover, consistent with previous findings,^17,18^ DPPG dispersions demonstrate a notably reversible thermal profile (Figure 2a,b). Remarkably, DPPG dispersions (Figure 2a) under conditions of low ionic strength exhibit a more cooperative excess heat peak compared to the peak observed in DPPG dispersions under physiological ionic strength condition (Figure 2b), as observed before.^17^ This cooperativity is evident in the smaller width at half-maximum (Δ*T*_1/2_), as detailed in Table 1, for more detail see Figure S2.

When considering the impact of the antibiotic on DPPG’s thermotropic behavior under both low and physiological ionic conditions, several noteworthy observations can be made. Focusing on low ionic conditions (Figure 2, left column), the presence of 10 mol % MFX (Figure 2c) and 20 mol % MFX (Figure 2e) results in a narrower transition of the gel–fluid peak compared to the transition observed in pure DPPG dispersions, particularly within the realm of exothermic processes (as indicated in Table 1). It is important to highlight that for endothermic processes, the thermal peaks with and without MFX are centered at the same temperature within standard deviation, as denoted by the dotted line in the left column of Figure 2.

DSC curves of DPPG in the presence of levofloxacin (LVX), a second-generation fluoroquinolone, indicate that LVX induces the formation of antibiotic-rich and antibiotic-poor regions within DPPG membranes.^17^ Interestingly, in the presence of moxifloxacin (MFX), the DPPG traces do not suggest the formation of such domains, as only a single thermal transition peak is observed. This behavior contrasts with that of LVX, which produces two distinct thermal transition peaks indicative of domain formation. These results suggest that MFX diffuses more uniformly into DPPG membranes than LVX, rather than remaining segregated in specific membrane regions.

Le-Deygen et al.^5^ investigated the structural changes induced by high concentrations of MFX in extruded lipid dispersions composed of pure DPPC and DPPC/cardiolipin (CL) mixtures (8:2 molar ratio), employing a lipid-to-antibiotic molar ratio close to 1:1. They demonstrated that MFX causes pronounced changes in the thermotropic behavior of zwitterionic DPPC dispersions, including the splitting of the main phase transition peak into two distinct events: one at a lower temperature (*T* = 33 °C) and another coinciding with the main transition of pure DPPC (≈41 °C) with a marked decrease in the intensity of the original DPPC transition peak was also observed. Interestingly, after 1 day of storage, the DSC trace of the MFX–DPPC system closely resembled that of pure DPPC, suggesting low affinity of MFX for zwitterionic membranes and progressive migration of the drug from the bilayer to the aqueous phase. In contrast, anionic dispersions composed of DPPC/CL exhibited a broad gel-to-fluid transition band centered around 30 °C. The presence of MFX caused a shift of this transition to slightly higher temperatures (by ∼3 °C) and resulted in a pronounced narrowing of the thermal peak. Similar behavior was observed in our study for DPPG dispersions interacting with MFX (Figure 2), suggesting that MFX interacts primarily at the level of the polar headgroups, promoting closer packing of the acyl chains and enhancing the cooperativity of the thermal transition. Notably, unlike the reversible behavior observed in the MFX-DPPC system, the DSC curves of the DPPC/CL-MFX system remained essentially unchanged after 24 h of storage, reinforcing the notion that MFX exhibits greater affinity for anionic membranes.^5^

#### 3.1.2 Electron Spin Resonance (ESR) with Spin Probes

Spin-label spectroscopy is a well-established method for assessing the structural and dynamic properties of lipid bilayer, see ref 14. This approach enables precise evaluation of lipid packing at the nanoscopic level surrounding the paramagnetic probe. Additionally, ESR spectra can yield insights into the local polarity in the immediate environment of the probe.^14,15^ In the present study, we evaluated membrane order/rigidity and polarity variations at distinct depths within ionic bilayers. Specifically, measurements were taken at the positions corresponding to the fifth and 16th carbon atoms of the lipid acyl chains, representing regions near the polar headgroups and the hydrophobic core of the bilayer, respectively. Considering that exogenous compounds may interact differently with membranes in gel and fluid states, we employed spin-label spectroscopy to examine the structural effects induced by MFX in anionic bilayers over a temperature range from 10 to 60 °C. This allowed us to probe both gel and fluid phases of dipalmitoyl lipid bilayers (see Figure 2).

Figure 3 displays the ESR spectra of the paramagnetic probes 5-PCSL (Figure 3a,c) and 16-PSCL (Figure 3b,d) incorporated into gel DPPG liposomes under low ionic strength conditions (Figure 3a,b) and physiological ionic strength conditions (Figure 3c,d). Figure 3 shows spin probes spectra in the absence (black lines) and the presence of increasing amounts of MFX, 10 mol % (red lines), and 20 mol % (blue lines).

**Figure 3:**
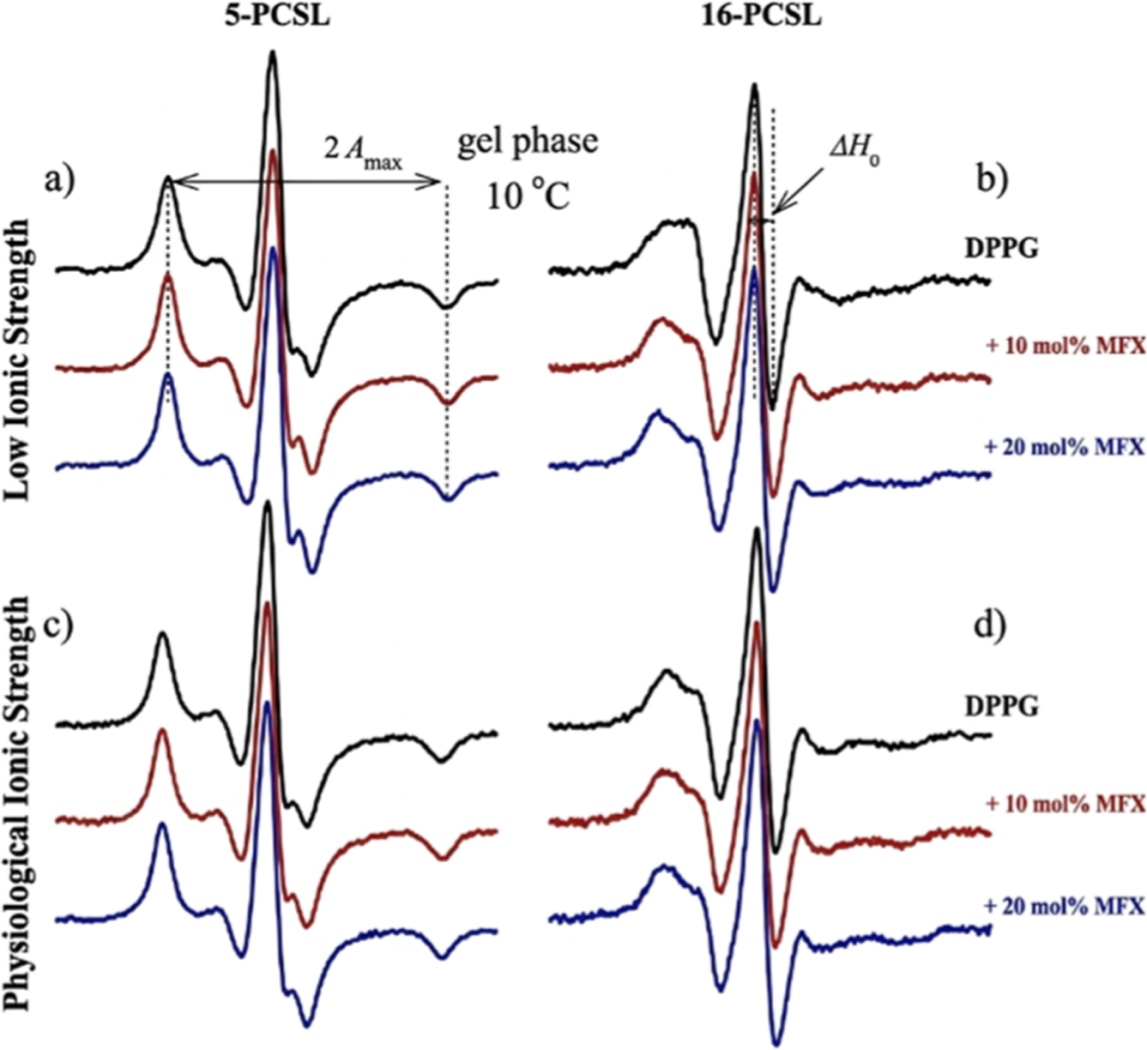
Typical ESR spectra of 5-PCSL (a,c) and 16-PCSL (b,d) incorporated into dispersions of gel (10 °C) DPPG vesicles in buffer at low (a,b) and physiological (c,d) ionic strength. The ESR spectra were obtained in the absence of MFX (black lines), in the presence of 10 mol % (red lines) and 20 mol % MFX (blue lines). The maximum hyperfine splitting (2 *A*_max_) and the central line width (Δ*H*_0_) are indicated. Total spectral width is 100 G.

As discussed before,^15^ empirical parameters measured directly from the ESR spectra provide information of the environment of the probe. For gel membranes, the maximum outer hyperfine splitting (2 *A*_max_, see Figure 3a,c) gives information about probe’s mobility. As higher 2 *A*_max_ values, higher are the restriction of the probe’s mobility Rozenfeld et al.^15^ Figure 4 displays the plot of 2 *A*_max_ measured directly from 5-PCSL spectra for low (Figure 4a) and physiological (Figure 4b) ionic strength conditions as a function of temperature. Pure DPPG dispersion at physiological ionic strength (Figure 4b) yields higher 2 *A*_max_ values than those observed for DPPG dispersions at low ionic strength (Figure 4a). This effect is likely due to the solvation layer of counterions that shield the anionic polar head of PG groups, thereby hindering the interaction between the acyl chain and increasing lipid packing. As expected, the increase in temperature induced relative fluidity, evidenced by the decrease in 2 *A*_max_ values with rising temperature, Figure 4.

**Figure 4:**
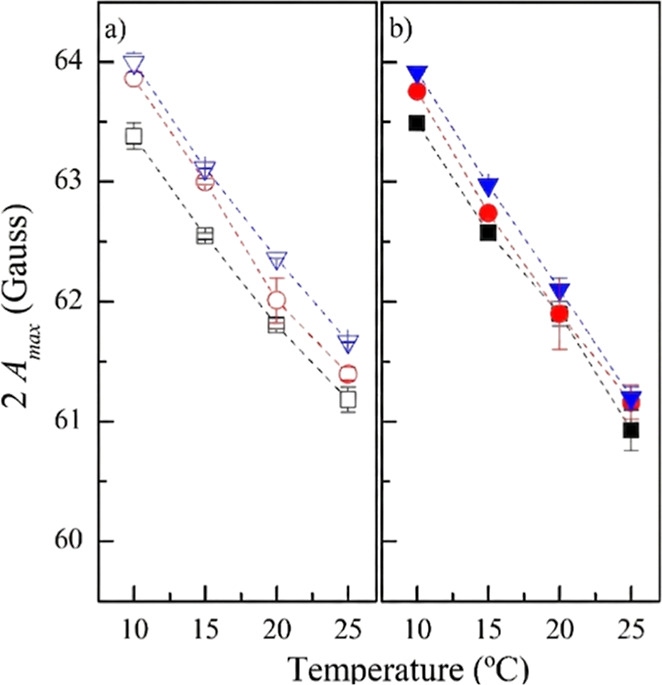
Temperature dependence of the outer hyperfine splitting (2 *A*_max_) measured on the ESR spectrum of 5-PSCL embedded into 3 mmol L^–1^ dispersions of gel DPPG liposomes in buffer at low (a) and physiological ionic strength (b). The data were obtained in the absence of MFX (black squares), in the presence of 10 mol % MFX (red circles), and 20 mol % MFX (down blue triangles). Error bar indicates standard deviation of at least three experiments with different samples. If not shown, the deviation was found to be smaller than the symbol.

Interestingly, increasing concentrations of MFX led to an enhancement of the 2 *A*_max_ values for DPPG dispersions under low and physiological ionic strength conditions (Figure 4), suggesting that the antibiotic promotes tighter lipid packing in gel-phase DPPG vesicles at the depth of the fifth carbon.

The inner core of the DPPG vesicles were monitor by the probe 16-PCSL. For gel vesicles, the parameter Δ*H*_0_ (Figure 3b) is effective in providing information about the motion of the 16-PCSL probe, as higher Δ*H*_0_ values indicate greater restriction of the probe’s motion. Figure 5 depicts Δ*H*_0_ as a function of the temperature for DPPG dispersion at low (Figure 5a) and at physiological (Figure 5b) ionic strength conditions at absence black squares and at the presence of 10 mol % (red circles) and 20 mol % MFX (blue triangles).

**Figure 5:**
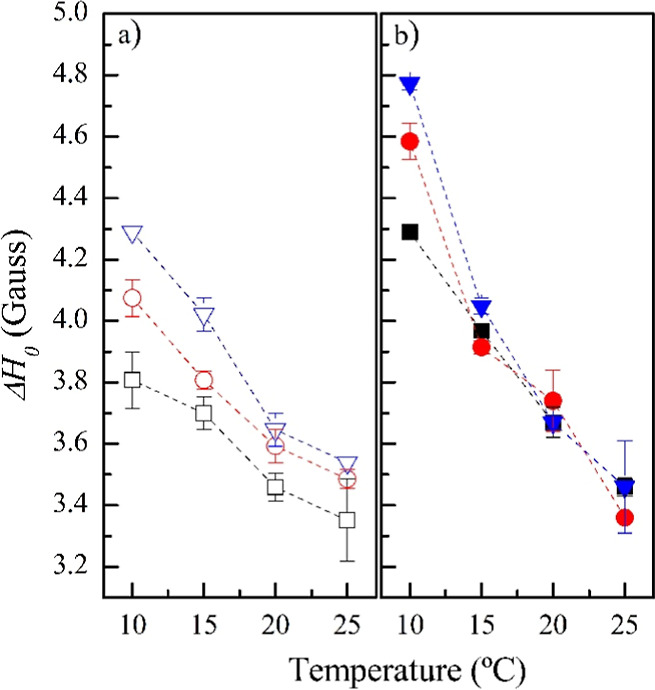
Temperature dependence of the central field line width (Δ*H*_0_) measured on the ESR spectrum of 0.3 mol % of 16-PCSL embedded into 3 mmol L^–1^ dispersions of gel DPPG liposomes in buffer at low (a) and at physiological ionic strength (b) in the absence of MFX (black squares), in the presence of 10 mol % MFX (red circles), and 20 mol % MFX (blue down triangles). Error bar indicates standard deviation of at least three experiments with different samples. If not shown, the error bars were found to be smaller than the symbol or equal to zero.

As expected, gel-phase of pure DPPG dispersions under low ionic strength conditions (Figure 5a) exhibit lower Δ*H*_0_ values compared to those observed at physiological ionic strength (Figure 5b). This result likely reflects a looser lipid packing in the membrane core, attributed to reduced shielding of the anionic headgroups under low ionic strength, which favors electrostatic repulsion between phosphatidylglycerol moieties. In contrast, at physiological ionic strength, the increased screening of the negative charges leads to diminished repulsion and, consequently, tighter membrane packing. The presence of 10 and 20 mol % MFX led to an increase in Δ*H*_0_, consistent with a more restricted motion of the 16-PCSL probe. This result indicates that the antibiotic promotes tighter lipid packing near the bilayer center in DPPG dispersions under low ionic strength (Figure 5a).

Figure 6 shows the ESR spectra of 5-PCSL (Figure 6a,c) and 16-PCSL (Figure 6b,d) incorporated into 3 mmol L^–1^ fluid-phase DPPG dispersions under low (Figure 6a,b) and physiological ionic strength conditions (Figure 6c,d). In all conditions, the spectra exhibit a single component, indicating that each spin label experiences a homogeneous environment within the bilayer, with no evidence of distinct populations or domains, either in the absence or in the presence of 10 and 20 mol % MFX.

**Figure 6:**
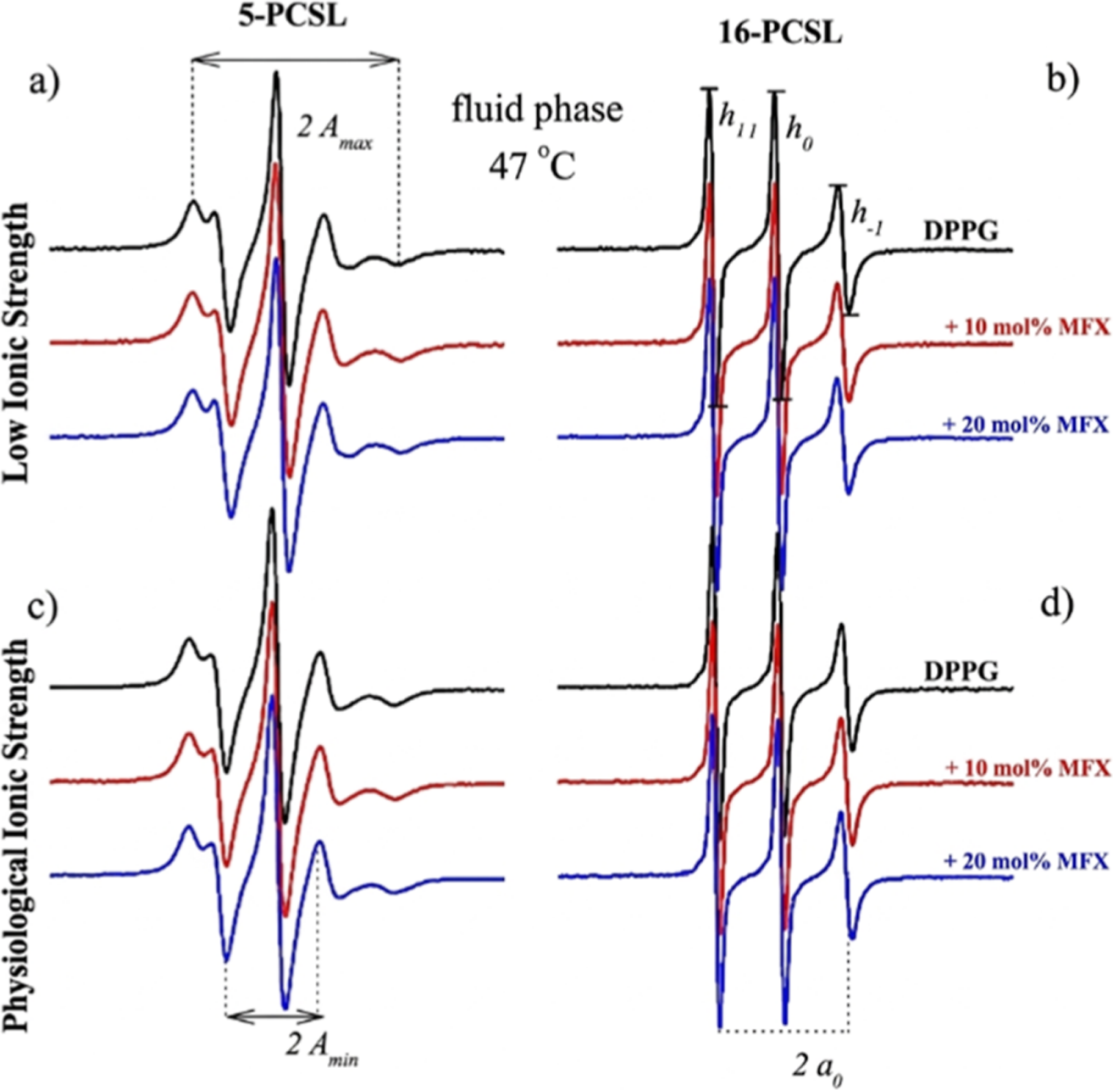
Typical ESR spectra of 5-PCSL (a,c) and 16-PCSL (b,d) incorporated into dispersions of fluid (47 °C) DPPG liposomes in buffer at low (a,b) and physiological (c,d) ionic strength. The ESR spectra were obtained in the absence of MFX (black lines), in the presence of 10 mol % (red lines) and 20 mol % MFX (blue lines). The maximum and minimum hyperfine splitting (2 *A*_max_ and 2 *A*_min_, respectively), the amplitudes of the low (*h*_+1_), central (*h*_0_) and high (*h*_–1_) fields and the isotropic hyperfine splitting (*a*_0_) are indicated. Total spectral width is 100 G.

To evaluate the effects of MFX on fluid-phase DPPG membranes, we determined the effective order parameter, *S*_eff_ (eq 3), which provides information about the lipid ordering and mobility specifically at the depth corresponding to the fifth carbon atom of the acyl chain. A *S*_eff_ value of 1 indicates that all spin labels are perfectly aligned parallel to the bilayer normal, reflecting a highly ordered environment at this position. Conversely, *S*_eff_ values near zero signify rapid, isotropic, and unrestricted probe motion, characteristic of a loosely packed membrane region at the fifth carbon level, see ref 18 and references therein. As expected, the DPPG dispersion under low ionic strength conditions displays lower *S*_eff_ values (Figure 7a) compared to DPPG under physiological ionic strength (Figure 7b), reflecting reduced shielding of the anionic headgroups and consequently increased electrostatic repulsion, which leads to a less tightly packed membrane. The presence of MFX under both low and physiological ionic strength conditions enhanced *S*_eff_ values, indicating that the antibiotic promotes membrane organization at the level of the fifth carbon. LVX was also found to organize DPPG membranes at the fifth carbon.^17^ Similarly, MFX has been reported to promote lipid packing near the interfacial region between the polar headgroups and the acyl chains in fluid-phase vesicles composed of DMPG under relatively high salt content ([NaCl] = 100 mmol L^–1^), as demonstrated by changes in bilayer organization monitored using 5-doxyl-stearic acid as a spin probe.^6^

**Figure 7:**
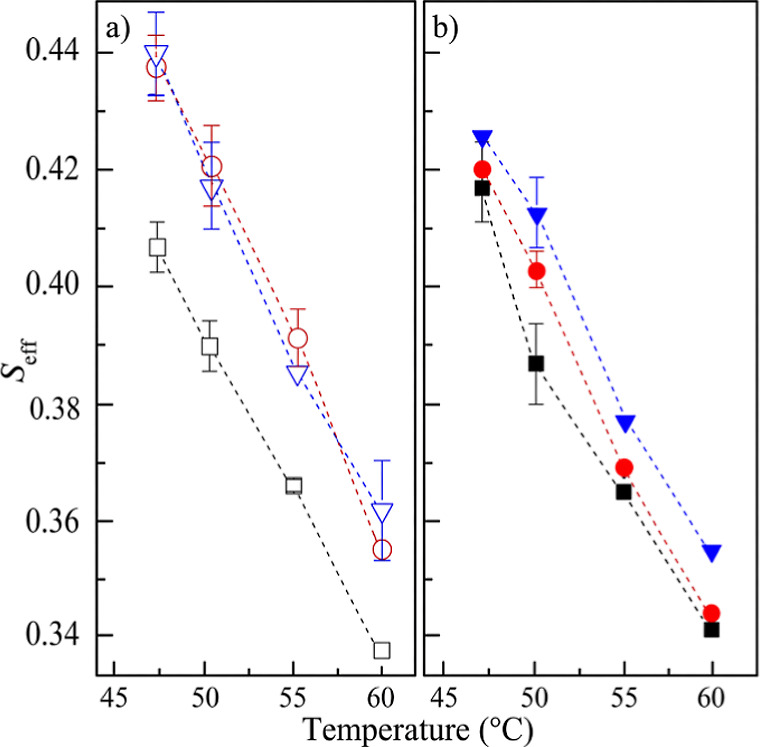
Temperature dependence of the effective order parameter (*S*_eff_), calculated from the ESR spectra of 5-PCSL incorporated into 3 mmol L^–1^ fluid DPPG liposomes under low (a) and physiological (b) ionic strength conditions. Data are shown for liposomes in the absence of MFX (black squares), and in the presence of 10 mol % MFX (red circles) and 20 mol % MFX (blue downward triangles). Open and solid symbols represent samples under low and physiological ionic strength conditions, respectively. Error bars correspond to the standard deviation from at least three independent experiments; when not visible, they are smaller than the symbol size.

In fluid-phase bilayers, the 16-PCSL spin label typically exhibits highly dynamic motion, enabling the direct determination of the amplitudes of its three hyperfine lines (*m*_*l*_ = +1, 0, −1) from the ESR spectra (Figure 8). The ratio of the high and central field lines amplitude (*h*_–1_/*h*_0_, see Figure 6b) serves as an empirical indicator of bilayer packing: as molecular motion becomes more rapid and disordered, this ratio approaches unity.^15^

**Figure 8:**
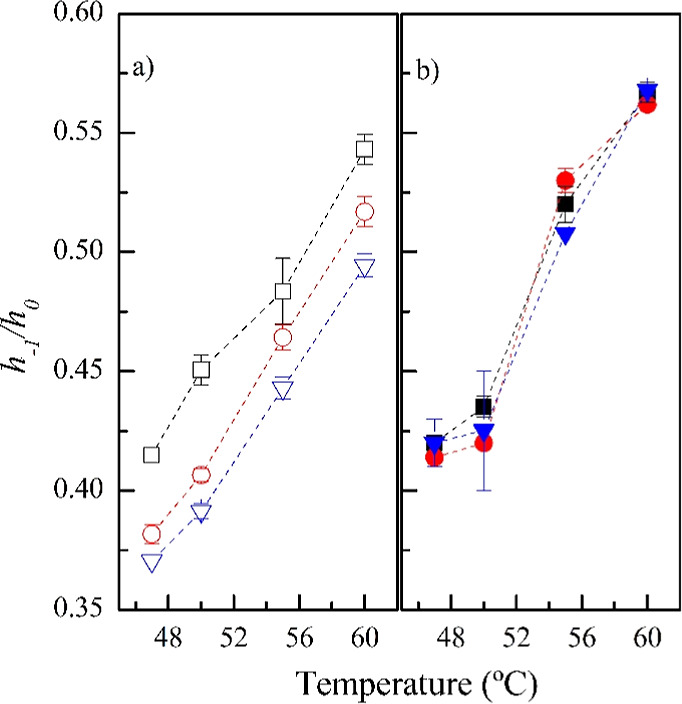
Temperature dependence of the ratio of the low and central field line amplitudes, *h*_–1_/*h*_0_, measured on the ESR spectrum of 0.3 mol % of 16-PCSL embedded into 3 mmol L^–1^ dispersions of DPPG liposomes in buffer at low (a) and physiological ionic strength (b). The data were obtained in the absence of MFX (black squares), and in the presence of 10 mol % MFX (red circles), and 20 mol % MFX (blue down triangles) Error bar indicates standard deviation of at least three experiments with different samples. If not shown, the error bars were found to be smaller than the symbol or equal to zero.

Figure 8 shows the ratio *h*_–1_/*h*_0_ as a function of temperature for fluid DPPG dispersions under low (Figure 8a) and physiological (Figure 8b) ionic strength conditions. As expected, the *h*_–1_/*h*_0_ values for pure DPPG at low salt concentration are higher than those observed at high salt concentration. This difference once again reflects the shielding effect of the increased concentration of counterions under physiological ionic strength, which screens the polar headgroups and promotes stronger tail-to-tail interactions, resulting in a more tightly packed bilayer region. Concerning the experiment conducted under low ionic strength conditions (Figure 8a), the addition of 10 and 20 mol % moxifloxacin (MFX) leads to a noticeable decrease in the *h*_–1_/*h*_0_ ratio, indicating that the antibiotic promotes tighter lipid packing within the hydrophobic core of the DPPG bilayer. In contrast, under physiological ionic strength conditions (Figure 8b), MFX induces only marginal changes; taking into account the associated error bars, these variations suggest that MFX does not significantly impact lipid packing in the DPPG bilayer core. Consistently, investigating the interaction of MFX with anionic DMPG dispersions at elevated salt concentrations, Neves et al.^6^ also reported no evidence of increased packing in the hydrophobic core of DMPG bilayers, as assessed using 16-doxyl stearic acid used as a spin probe.

The isotropic hyperfine splitting constant (*a*_0_) provides valuable information about the local polarity of the nanoregion of the spin probe, in the present case, the membrane core. Higher *a*_0_ values are associated with increased polarity, resulting from water penetration into this otherwise hydrophobic region.^15^ Thus, variations in *a*_0_ may reflect changes in membrane structure resulting from lipid–molecule interactions, which can affect the hydration of the lipid bilayer. Figure 9 shows *a*_0_ values, measured directly from the 16-PCSL spectra, as a function of temperature under low (Figure 9a) and physiological (Figure 9b) ionic strength conditions, in the absence and presence of increasing MFX concentrations. The *a*_0_ values for pure DPPG dispersions are comparable under both conditions. Upon MFX addition, *a*_0_ values decrease across all temperatures and ionic strengths (Figure 9a,b), indicating reduced local polarity and dehydration of the bilayer core induced by the antibiotic.

**Figure 9:**
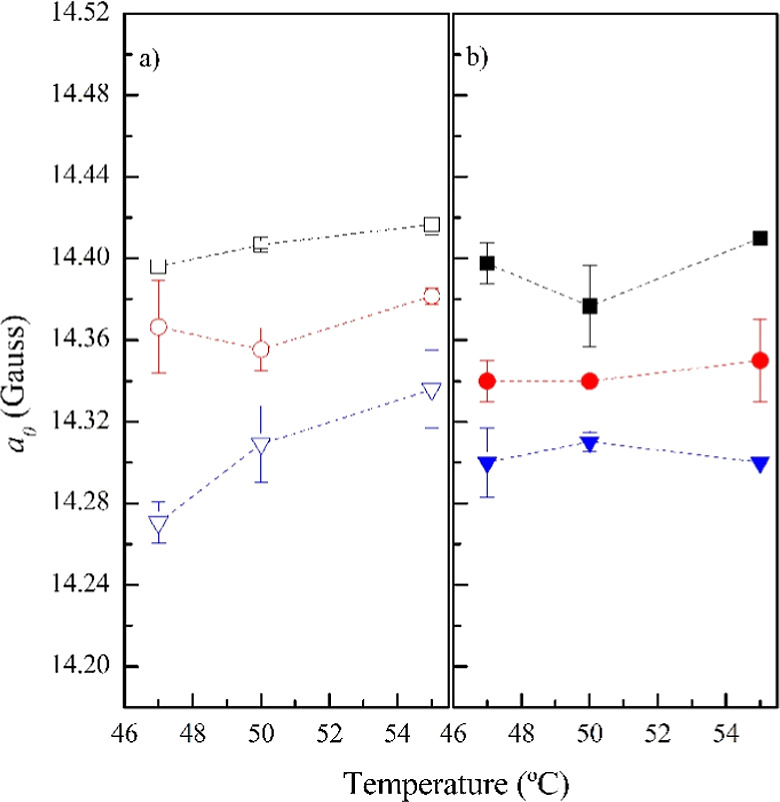
Temperature dependence of the isotropic hyperfine splitting (*a*_0_) measured on ESR spectra of 0.3 mol % of 16-PCSL incorporated into 3 mmol L^–1^ dispersions of fluid (47 °C) DPPG liposomes in buffer at low (a) and physiological ionic strength (b). The data were obtained in the absence of MFX (black squares), in the presence of 10 mol % MFX (red circles), and 20 mol % MFX (blue down triangles). Error bar indicates standard deviation of at least three experiments with different samples. If not shown, the error bars were found to be smaller than the symbol or equal to zero.

### 3.2 Intrinsic Fluorescence Emission of MFX: Modifications Induced by Anionic Membranes

As with most fluoroquinolones (FQs), MFX is naturally fluorescent. FQ fluorescence emission is highly sensitive to the fluorophore structure and its surrounding environment.^12,29,30^ Hence, we exploited MFX fluorescence to investigate its interactions with DPPG vesicles at low (Figure 10a,c) and physiological ionic strength (Figure 10b,d), obtaining information about the antibiotic’s structure. At physiological pH (7.4), MFX displays a maximum emission at 466 nm (Figure 10), in agreement with previous reports.^5,31,32^ The increase in gel (Figure 10a) and fluid (Figure 10c) DPPG liposome concentration under low ionic strength conditions gradually induces a significant fluorescence redshift, as shown in Figures 10a,c and 11a. In contrast, the presence of either gel or fluid liposomes under physiological ionic strength conditions causes only a minor redshift of MFX emission spectra, see Figures 10b,c and 11b.

**Figure 10:**
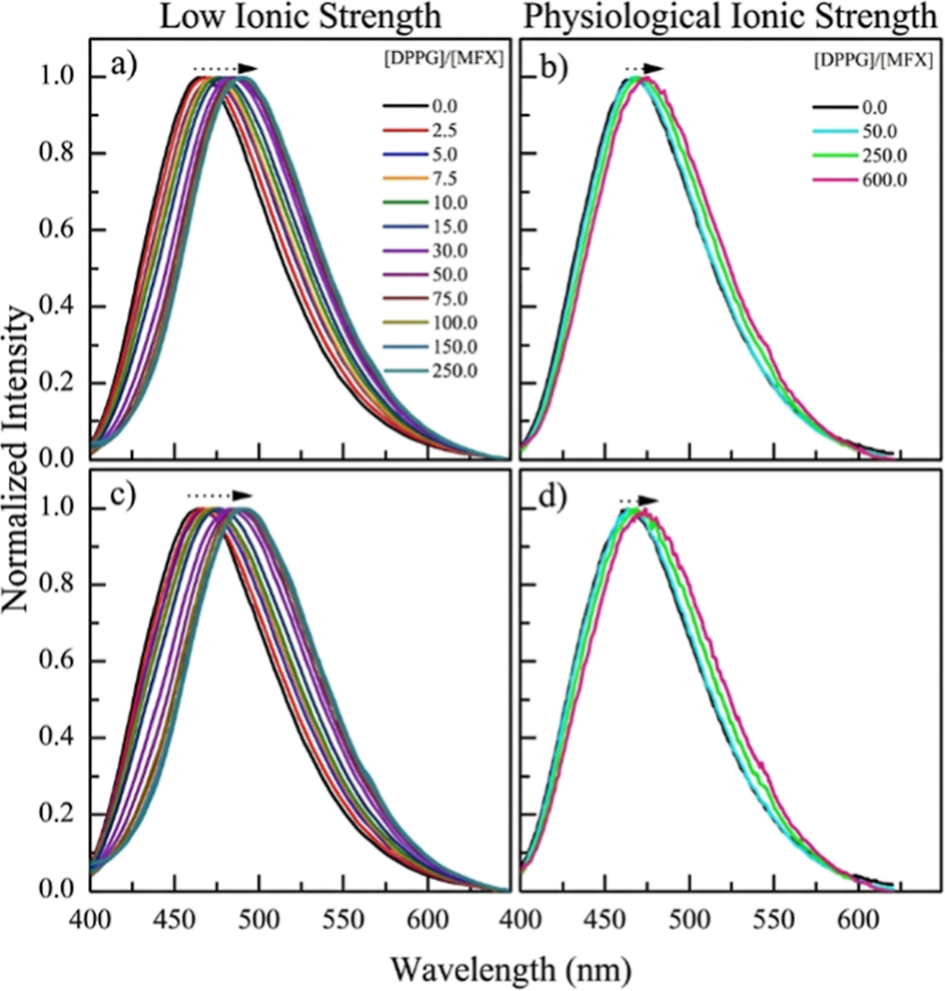
Emission spectra of MFX (8 μmol L^–1^) in buffer at low (a,c) and physiological (b,d) ionic strength in the absence and in the presence of increasing amounts of DPPG vesicles. Excitation light beam at 345 nm.

**Figure 11:**
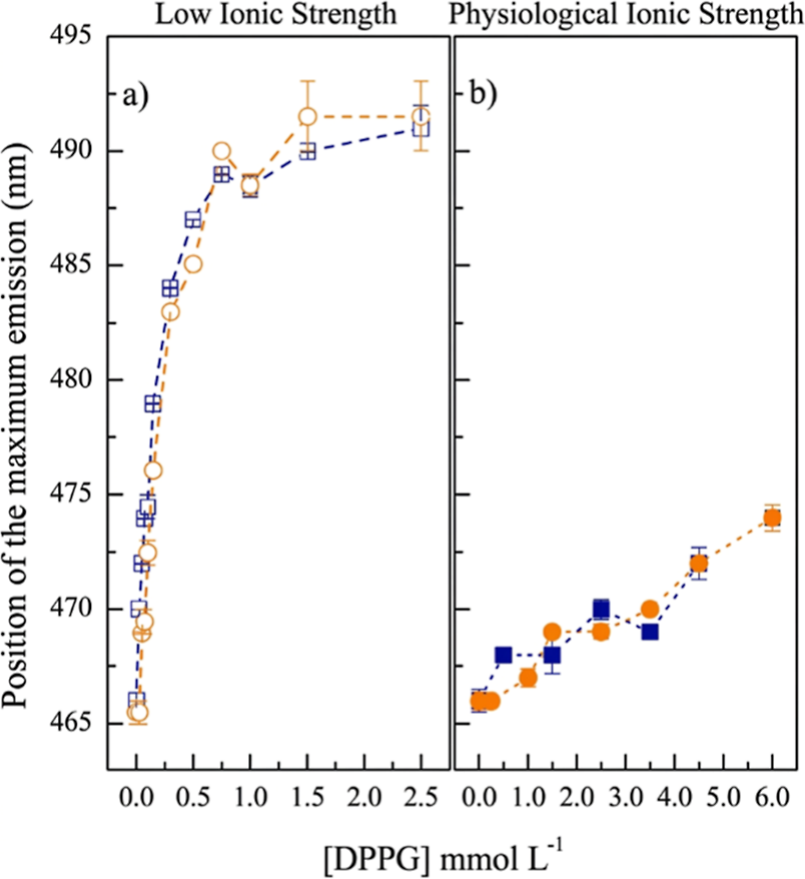
Position of the maximum emission of MFX in buffer at low (a) and physiological (b) ionic strength as a function of the concentration of gel (25 °C, blue squares) and fluid (50 °C, orange circles) DPPG vesicles.

Fluorophores typically exhibit a relative blue shift in their fluorescence spectra upon binding to lipid bilayers, reflecting the membrane’s dehydrated environment and the consequent reduction in dipolar relaxation rate.^33^ In our case (Figures 10 and 11), as observed for many FQs binding to anionic structures,^12,17,34^ a relative red-shift occurs, likely due to the lower local pH around the anionic amphiphilic aggregate, in agreement with the Gouy–Chapman model.^35^ Note that the emission of MFX under acidic conditions (low pH) occurs at longer wavelengths than that observed at physiological pH 7.4, see Figure S3. Therefore, the observed red shift in fluorescence arises from the combined effects of the transition from the zwitterionic to the cationic form of MFX and the concomitant decrease in polarity in the vicinity of the vesicle surface.^12^

To further support this interpretation, plotting the fluorescence intensity at 460 nm as a function of buffer pH (Figure S3) yielded two apparent p*K*_a_ values (p*K*_a1_ = 5.8 ± 0.1 and p*K*_a2_ = 9.91 ± 0.05), corresponding to the cationic–zwitterionic and zwitterionic–anionic equilibria of MFX, respectively. These values are close to those reported in the literature.^36^

It is worth noting that the red-shift observed for the interaction with DPPG dispersions at low ionic strength is about 27 nm (Figure 11a), compared to only 8 nm under physiological ionic strength conditions (Figure 11b). This is likely due to the reduction of the surface potential of PG vesicles under high salt conditions^37^ strongly indicating that the interaction between MFX and DPPG lipids is highly dependent on electrostatic interactions. Moreover, it is very interesting that the observed red-shift is the same considering the error bars for gel (25 °C) or fluid (50 °C) vesicles either under low and high salt concentration. Assuming a temperature-independent surface potential and applying the Gouy–Chapman model (eq 5), for a surface potential ranging from −100 mV to −10 mV, we calculated that the pH ratio around the PG vesicles between 25 and 50 °C is very close to unity, indicating that the surface pH around the vesicles remains nearly unchanged when the temperature increases from 25 to 50 °C.

5 $${\left[{H_{3}O}^{+}\right]}_{surf}={\left[{H_{3}O}^{+}\right]}_{bulk}e^{-q\psi /k_{B}T}$$

$$pH_{surf}={pH}_{bulk}+0.434\frac{q\psi }{k_{B}T}$$

In these expressions [H_3_O^+^]_surf_ and [H_3_O^+^]_bulk_ denote the hydronium concentrations at the vesicle surface and in the bulk phase, respectively; pH_surf_ and pH_bulk_ refer to the corresponding pH values; *q* is the elementary charge; ψ is the vesicle surface potential; *k*_B_ is the Boltzmann constant; and *T* is the absolute temperature.^12^

This is may account for the observed red shift and suggests that MFX has comparable affinity for both gel-phase and fluid-phase PG membranes. Taking into account that the antibiotic, once zwitterionic, becomes cationic, it is highly probable that MFX is located close to the vesicle surface.

Fluorescence spectra can be significantly affected by light scattering, often caused by liposomes. Although the presented spectra were corrected by using eq 2, the use of a complementary technique was considered important. Consequently, time-resolved fluorescence was employed, a method minimally influenced by light scattering,^21,33^ allowing the determination of excited-state lifetimes through analysis of fluorescence decay curves. Figure 12 shows the fluorescence decay curves of MFX in the absence and in the presence of DPPG dispersions under low (Figure 12a,c) and physiological (Figure 12b,d) ionic strength, with the liposomes in the gel phase (25 °C, Figure 12a,b) and fluid phase (50 °C, Figure 12c,d).

**Figure 12:**
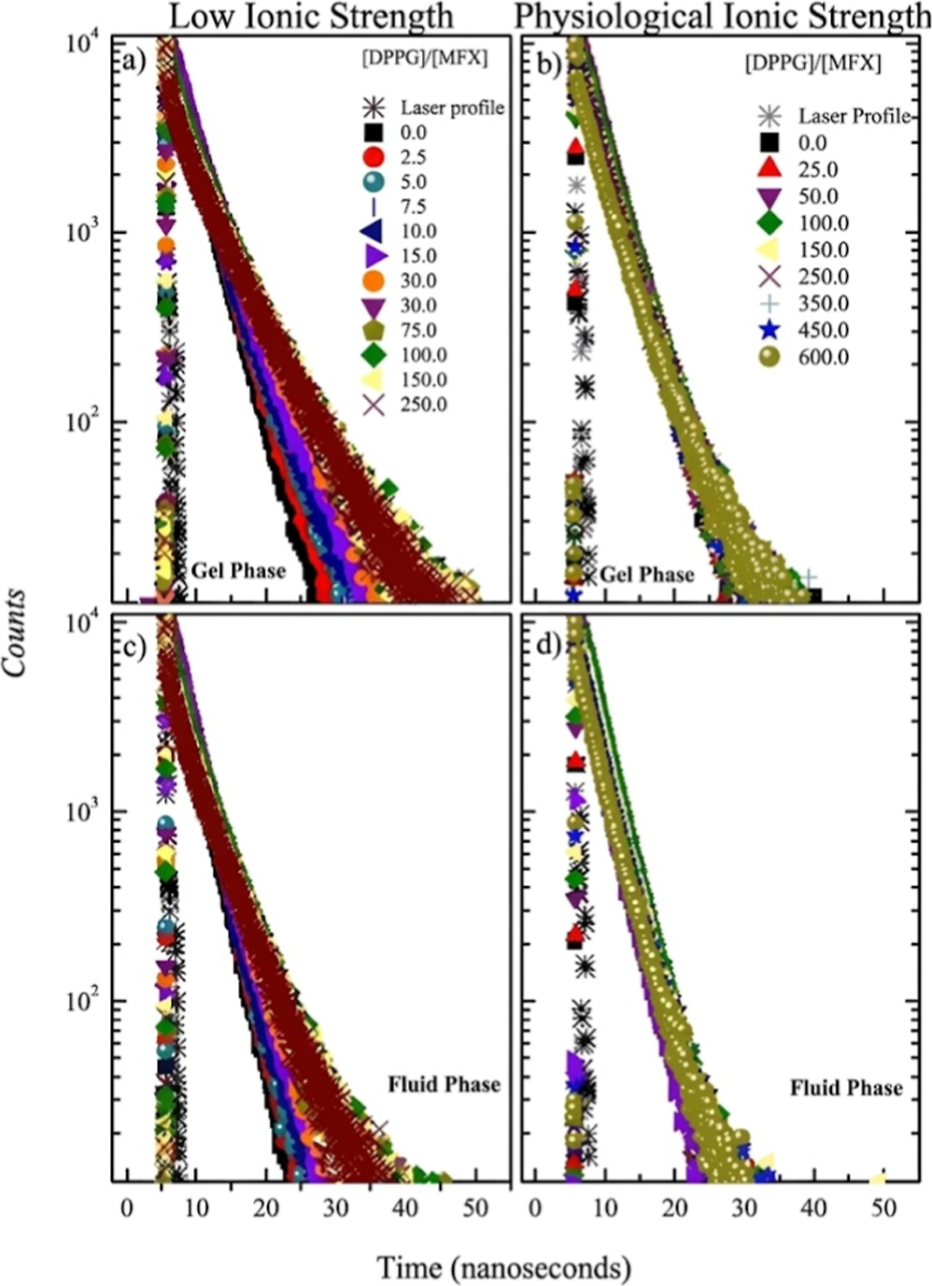
Typical fluorescence decay curves of MFX in buffer at low (a,c) and physiological (b,d) ionic strength in the presence of increasing concentrations of gel vesicles (25 °C) of DPPG (a,b) and fluid vesicles (50 °C) of DPPG. Excitation light beam at 284 nm and emission at 466 nm.

MFX fluorescence decay curves, both in the absence and in the presence of liposomes, were well fitted by a biexponential model, according to statistical criteria, yielding two fluorescence lifetimes, a short lifetime (τ_1_) and a long lifetime (τ_2_). At 25 °C, τ_1_ = (2.65 ± 0.08) ns and τ_2_ = (3.5 ± 0.1) ns were found. As expected, increasing the temperature to 50 °C led to a reduction in the fluorescence lifetimes due to the enhancement of nonradiative decay rates, with the observed values being τ_1_ = (1.4 ± 0.2) ns and τ_2_ = (2.80 ± 0.04) ns.

Figure 13 shows the long lifetime of MFX (τ_2_) as a function of DPPG dispersions in the gel phase (25 °C; blue downward triangles) and fluid phase (50 °C; orange squares) under low ionic strength (Figure 13a) and physiological ionic strength (Figure 13b) conditions. Under low ionic strength, the long lifetime of MFX increased monotonically in the presence of DPPG dispersions, allowing the data to be fitted using eq 4 and enabling the determination of distinct partition constants: *K*_p_ = (2.7 ± 0.2) × 10^4^ for gel membranes (25 °C) and *K*_p_ = (1.4 ± 0.1) × 10^4^ for fluid membranes (50 °C). The values obtained suggest that MFX exhibits very similar affinity for both gel and fluid DPPG membranes. Additionally, as indicated by steady-state and time-resolved fluorescence, increasing the ionic strength reduces the affinity of the antibiotic for anionic membranes. Under physiological salt conditions, the lifetimes (Figure 13b) do not reach saturation, indicating that most MFX molecules remain in the aqueous environment rather than binding to the liposomes. This strongly suggests that the interaction is highly dependent on electrostatic attraction between the antibiotic and the lipid polar headgroups.

**Figure 13:**
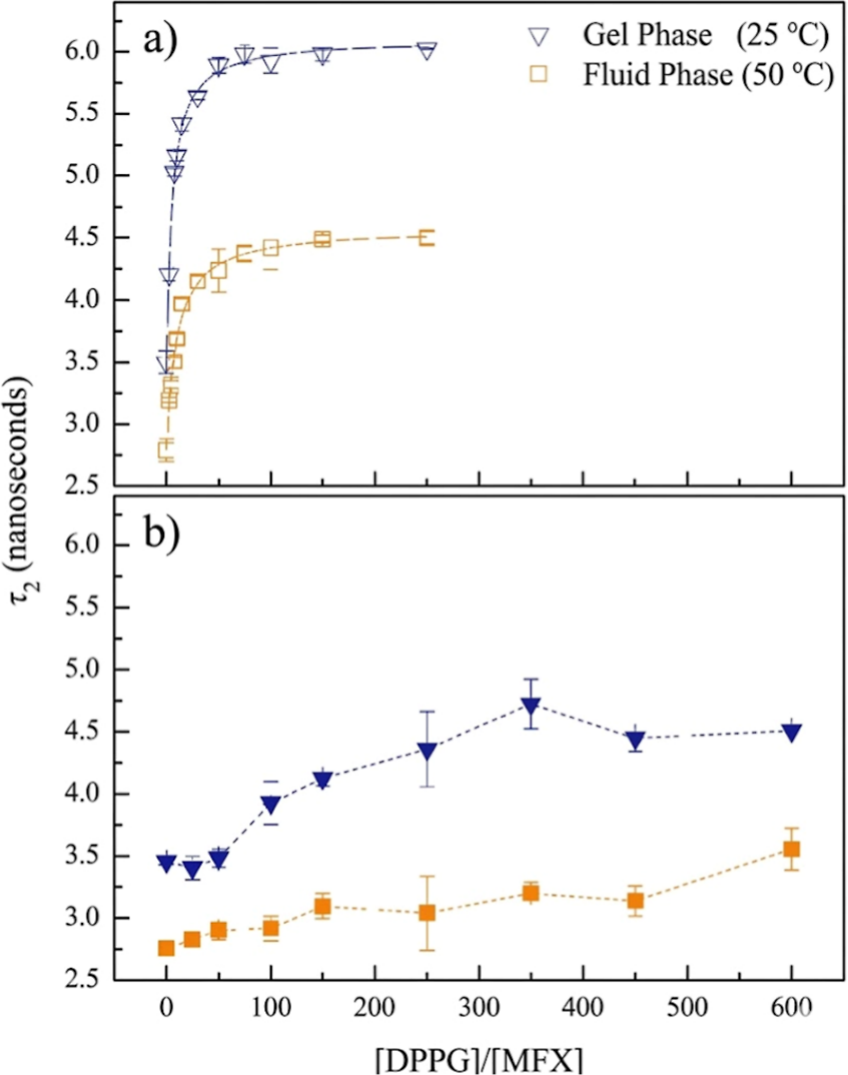
Longer fluorescence lifetime of MFX (τ_2_) in buffer at low (a) and physiological (b) ionic strength as a function of gel (25 °C) vesicles (blue squares) and fluid (50 °C) vesicles (orange circles) of DPPG. The continuous lines consist in the data fitting by using eq 4. The dotted lines are only guide for the eyes.

Interestingly, MFX exhibits a partition constant (*K*_p_) that is 2 orders of magnitude higher than that of LVX for DPPG membranes under very similar conditions, indicating that MFX is more lipophilic than LVX.^17^ This is particularly relevant given that MFX does not depend on protein channels in the bacterial membrane to access the cytosol, unlike LVX, which requires porin-mediated uptake.

## 4 Conclusions

MFX significantly modulates the physicochemical properties of anionic DPPG membranes, with effects consistently observed under both gel and fluid phases. DSC traces revealed that MFX narrows the gel-to-fluid phase transition peak, indicating enhanced cooperativity of lipid packing without evidence of domain separation. ESR spectroscopy corroborated these findings, showing that MFX promotes tighter lipid organization both near the interfacial region and in the bilayer core, accompanied by a decrease polarity in the core of the anionic liposomes. Fluorescence experiments further confirmed that MFX associates strongly with DPPG membranes under low ionic strength, with partition constants on the order of 10^4^, for liposomes in both configurations: in the gel and fluid phases. Whereas physiological ionic strength markedly reduces this affinity, emphasizing the electrostatic nature of the interaction MFX-DPPG. Altogether, the data indicate that MFX preferentially locates at the lipid–water interface, where protonation driven by the negatively charged environment may occur, thereby altering the antibiotic’s structure. Given that membrane interactions are critical to antimicrobial activity, toxicity, and drug delivery, these results provide important physicochemical insights into the behavior of MFX in lipid environments and may guide the rational design of lipid-based carriers and the optimization of fluoroquinolone antibiotics.

## Supplementary Material



## Notes

Hydrodynamic diameter (*D*_z_) and polydispersity index (PDI) data for DPPG dispersions under different ionic strengths, temperatures, and MFX concentrations; fluorescence spectra of MFX at different excitation wavelengths; thermodynamic parameters extracted from DSC endothermic curves; pH-dependent fluorescence spectra and nonlinear fitting analysis using the modified Henderson–Hasselbalch equation (PDF)
