# Supplementary material for: Physicochemical Characterization of Anionic Lipid Membranes under Low and Physiological Ionic Strength: Effects of Moxifloxacin Assessed by Calorimetry, Spin-Label, Steady-State, and Time-Resolved Fluorescence
Source: ACS Omega. 2026 Jun 10;11(24):35112–23. doi: 10.1021/acsomega.5c11663 (PMC13295017; doi:10.1021/acsomega.5c11663)

# Supporting Information

# Physicochemical Characterization of Anionic Lipid Membranes under Low and Physiological Ionic Strength: Effects of Moxifloxacin Assessed by Calorimetry, Spin-label, Steady-State and Time-Resolved Fluorescence

Arthur Henrique Barrios Solano[b], Mariana C. Souza[b], Carla C. V. Medeiros[a], Arthur S. Borges[a], Bruno Sugamosto[a], Evandro L. Duarte[b], M. Teresa Lamy[b], and Gabriel S. Vignoli Muniz*[a,b]

[a]Instituto de Química, Universidade de Brasília, CEP 70910-900, Campus Universitário Darcy Ribeiro, Brasília, Brasil

[b]Instituto de Física, Universidade de São Paulo, CEP 05508-090, Cidade Universitária, São Paulo, Brasil

*Corresponding author: Gabriel S. Vignoli Muniz (gvignoli@usp.br)

**Table of contents**

**Table S1.** Hydrodynamic diameter ($D_z$) and polydispersity index (PDI) of DPPG dispersions (3 mmol $L^{-1}$) in the gel phase (25 °C) and fluid phase (60 °C), measured by dynamic light scattering (DLS), in buffer (10 mmol $L^{-1}$ HEPES, 1 mmol $L^{-1}$ EDTA, pH 7.4) under low ionic strength ([NaCl] = 3 mmol $L^{-1}$) and physiological ionic strength ([NaCl] = 150 mmol $L^{-1}$), after 31 extrusion cycles, in the absence and presence of different concentrations of MFX. **S3**

**Fig. S1.** Fluorescence spectra of MFX in buffer (10 mmol $L^{-1}$ HEPES, 3 mmol $L^{-1}$ NaCl, 1 mmol $L^{-1}$ EDTA, pH 7.4), excited at different wavelengths. **S4**

**Fig. S2** Thermodynamic parameters extracted from DSC endothermic curves: (a) temperature width at half maximum of the transition peak ($\Delta T_{1/2}$), main phase transition temperature ($T_m$), and (c) enthalpy change ($\Delta H$). **S5**

**Fig. S3** (a) Selected fluorescence spectra at different pH values. (b) Fluorescence intensity at 460 nm as a function of pH. Solid lines represent nonlinear fits using the modified Henderson–Hasselbalch equation.

$$I = \frac{I_1 10^{\mathrm{p}Ka} + I_2 10^{\mathrm{pH}}}{10^{\mathrm{pH}} + 10^{\mathrm{p}Ka}}$$

where $I_1$and $I_2$are the fluorescence intensities at 460 nm of the cationic and zwitterionic, or zwitterionic and anionic, MFX species. **S6**

**Table S1.** Hydrodynamic diameter ($D_z$) and polydispersity index (PDI) of DPPG dispersions (3 mmol $L^{-1}$) in the gel phase (25 °C) and fluid phase (60 °C), measured by dynamic light scattering (DLS), in buffer (10 mmol $L^{-1}$ HEPES, 1 mmol $L^{-1}$ EDTA, pH 7.4) under low ionic strength ([NaCl] = 3 mmol $L^{-1}$) and physiological ionic strength ([NaCl] = 150 mmol $L^{-1}$), after 31 extrusion cycles, in the absence and presence of different concentrations of MFX.

| Sample | [NaCl] (mmol $L^{-1}$) | Temp. ($^{o}$C) | Z-ave (nm) | PDI |
|---|---|---|---|---|
| | | **Gel phase** | | |
| DPPG | 3 | 25 | 112 ± 1 | 0.04 ± 0.02 |
| DPPG | 150 | 25 | 111 ± 2 | 0.07 ± 0.02 |
| + 10 mol% MOX | 3 | 25 | 116 ± 1 | 0.09 ± 0.02 |
| + 10 mol% MOX | 150 | 25 | 113 ± 1 | 0.07 ± 0.03 |
| + 20 mol% MOX | 3 | 25 | 119 ± 2 | 0.10 ± 0.02 |
| + 20 mol% MOX | 150 | 25 | 113 ± 2 | 0.07 ± 0.04 |
| | | **Fluid Phase** | | |
| DPPG | 3 | 60 | 120 ± 1 | 0.05 ± 0.02 |
| DPPG | 150 | 60 | 119 ± 2 | 0.09 ± 0.02 |
| + 10 mol% MOX | 3 | 60 | 121 ± 2 | 0.07 ± 0.03 |
| + 10 mol% MOX | 150 | 60 | 121 ± 2 | 0.10 ± 0.03 |
| + 20 mol% MOX | 3 | 60 | 120 ± 1 | 0.07 ± 0.02 |
| + 20 mol% MOX | 60 | 60 | 121 ± 3 | 0.10 ± 0.04 |

**Fig. S1.** Fluorescence spectra of MFX in buffer (10 mmol $L^{-1}$ HEPES, 3 mmol $L^{-1}$ NaCl, 1 mmol $L^{-1}$ EDTA, pH 7.4), excited at different wavelengths.

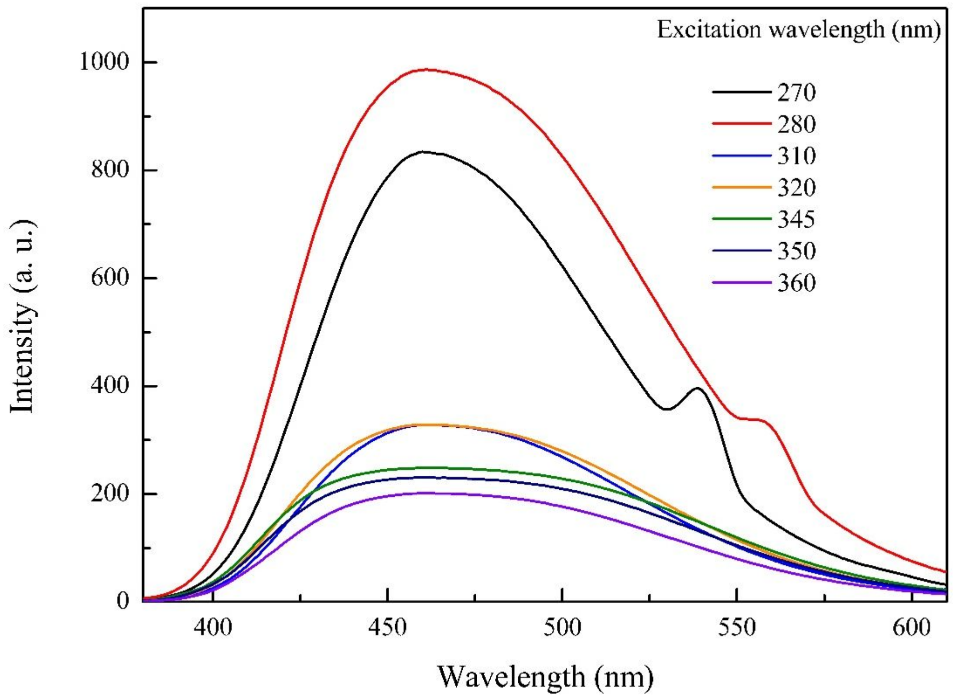

**Fig S2** Thermodynamic parameters extracted from DSC endothermic curves: (a) temperature width at half maximum of the transition peak ($\Delta T_{1/2}$), main phase transition temperature ($T_m$), and (c) enthalpy change ($\Delta H$).

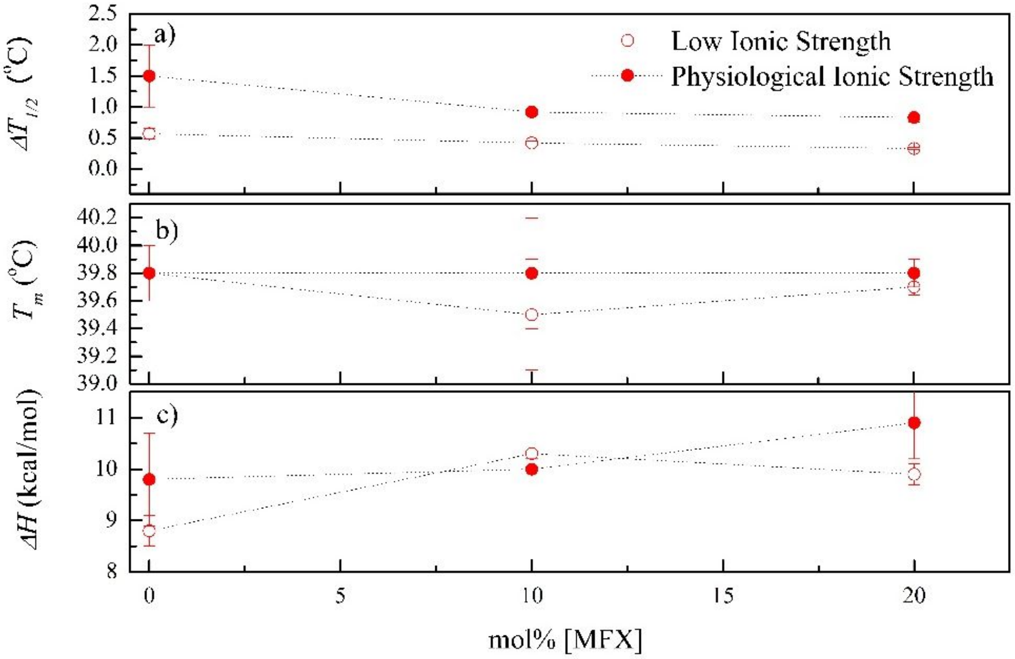

**Fig. S3** (a) Selected fluorescence spectra at different pH values. (b) Fluorescence intensity at 460 nm as a function of pH. Solid lines represent nonlinear fits using the modified Henderson–Hasselbalch equation.

$$I = \frac{I_1 10^{\mathrm{p}Ka} + I_2 10^{\mathrm{pH}}}{10^{\mathrm{pH}} + 10^{\mathrm{p}Ka}}$$

where $I_1$ and $I_2$ are the fluorescence intensities at 460 nm of the cationic and zwitterionic, or zwitterionic and anionic, MFX species.

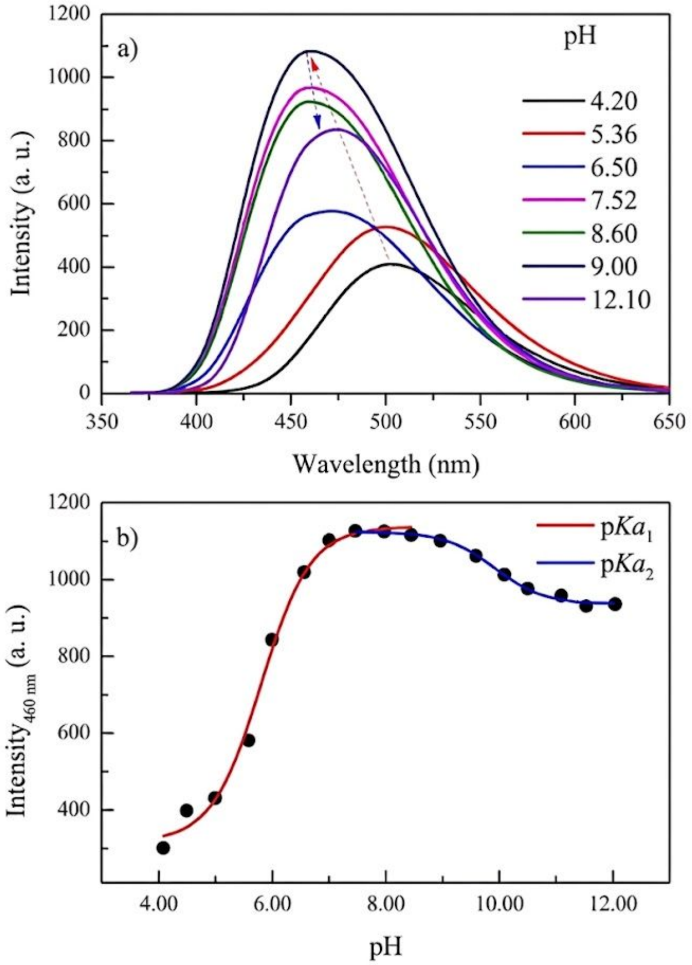

Supplement: Supplementary file 1 [file ao5c11663_si_001.pdf]
